# Commentary: Social stress contagion in rats: Behavioral, autonomic and neuroendocrine correlates

**DOI:** 10.3389/fnbeh.2017.00175

**Published:** 2017-09-19

**Authors:** Tony W. Buchanan, Stephanie D. Preston

**Affiliations:** ^1^Department of Psychology, Saint Louis University St. Louis, MO, United States; ^2^Department of Psychology, University of Michigan Ann Arbor, MI, United States

**Keywords:** empathy, social stress, emotional contagion, glucocorticoids, autonomic nervous system

Social animals can catch the emotional states of others. Feelings of elation spread through a crowd at a sporting event. Stressful experiences may be transmitted through a social network of mutual friends. Such contagious stress extends beyond our subjective feeling states to affect our heart rates or even the release of hormones. Many studies have demonstrated such “contagious stress” among humans, nonhuman primates, as well as rodents and other species (see de Waal and Preston, 2017). New work by Carnevali et al. (2017) at the University of Parma, Italy provides new clues about how the stress of those around us may affect our physiology and behavior.

Rats are social creatures whose physiology and behaviors are influenced by the state of other rats; such responses are thought to be adaptive for group survival. The observation of one individual under stress may indicate a threat, so other rats may benefit from noticing and responding accordingly. A number of studies have documented contagious responses to pain and fear in mice, rats, and humans, but not much work has focused on the contagion of social stress.

To address this issue, Carnevali et al. examined the contagion of social stress on both the animal that experienced the stress first hand as well as that animal's cagemate, who did not observe the cause of the stress. To do this, a “demonstrator” rat was paired up with an “observer” rat. The demonstrator rat was removed from the cage and underwent social defeat stress, in which the demonstrator (in this case, a male Wistar rats weighing between 300 and 350 g) was put in a cage with a much larger 500–600 g rat, who attacks the demonstrator rat. Social defeat paradigms effectively produce a stress response in the demonstrator rats, rats, including increased heart rate, the release of corticosterone, as well as social avoidance behavior.

Next, Carnevali et al. asked whether the stress experienced by the demonstrator rat would influence the physiology and behavior of the demonstrator's cagemate. To test this, the authors returned the demonstrator rat to their original cage with the observer rat. The same pattern of increased heart rate and corticosterone as well as increased social avoidance behavior was documented in the observer rats as it was in the demonstrator rats. Importantly, this “stress contagion” occurred despite the fact that the observer rats had not seen or heard the social defeat experience of the demonstrator rats (the social stress occurred in another, sound-attenuated room).

Upon the demonstrator rats' return to the original cage, the cagemate observer rats were able to detect the stress of the demonstrator, resulting in a vicarious stress response. The authors carefully ruled out the potential for olfactory signals from the attacker rat to influence responses of observers by showing that exposure to the bedding from the cage of the attacker rat did not elicit physiological changes in the observers in the absence of the demonstrator rat. However, olfactory, visual, and auditory signals from the demonstrator rat surely play a role in producing these contagious stress responses under normal circumstances. Future work should address just what these signals are that allow the observer rats to respond to the aftermath of stress in their cagemates. This work is novel in showing that stress occurring out of sight (indeed outside of immediate hearing and smell range) can be contagious between individuals.

Previous research has demonstrated similar patterns of social transmission of behavioral states in rodents (Knapska et al., 2006). Specifically, socially transferred fear responses have been demonstrated in a similar paradigm in which demonstrator animals underwent fear conditioning before being reunited with an observer animal. In this study, the observer showed greater startle reactivity and amygdala activation in response to their fear-conditioned cagemate. The work of Carnevali et al. extend this finding to a stress manipulation and to peripheral measures of stress reactivity (heart rate and corticosterone) that may have lasting effects on mental and physical health.

Previous work in humans has demonstrated the social contagion of stress, due to the direct observation of another under stress (Buchanan et al., 2012; Engert et al., 2014) as well as in response to stressed individuals in the aftermath of stress (Waters et al., 2014). Waters et al. (2014) subjected human mothers to a social stressor in a separate room from their babies. Upon their reunion with their stressed mothers, babies showed increased heart rates and increased social avoidance compared to babies in a control condition. The similarity between the results of Carnevali et al. with this work on stressed mothers and babies demonstrates cross-species congruence and the power of stress to affect those around us across space and time.

## Author contributions

TB wrote the first draft of this commentary. SP edited and contributed to the writing of the final draft.

### Conflict of interest statement

The authors declare that the research was conducted in the absence of any commercial or financial relationships that could be construed as a potential conflict of interest.
